# Supplementary material for: The efficacy of T790M mutation testing in liquid biopsy—Real clinic data
Source: PLoS One. 2022 May 6;17(5):e0267846. doi: 10.1371/journal.pone.0267846 (PMC9075651; doi:10.1371/journal.pone.0267846)
Supplement: S1 File — (DOCX) [file pone.0267846.s001.docx]

| Number | Presence of T790M mutation in *EGFR* gene (cfDNA) | Number of liquid biopsies | Tumor size (cT) | Lymph nodes metastases (cN) | Distant metastases | Sum of target lesions (mm) | Type of primary *EGFR* gene mutations | Generation of EGFR TKIs | Type of EGFR TKIs | Median PFS at the time of blood collection | Median PFS at the time of progression according to RECIST | Censored data |
| --- | --- | --- | --- | --- | --- | --- | --- | --- | --- | --- | --- | --- |
| 1 | Yes | 2 | 4 | 0 | Yes | 25 | Exon 19 deletion | 2 | Afatinib | 14 | 23 | No |
| 2 | Yes | 2 | 4 | 0 | No | 25 | L858R substitution | 2 | Afatinib | 17 | 33 | No |
| 3 | Yes | 1 | 4 | 0 | No | 9 | L858R substitution | 2 | Afatinib | 33 | 48 | Yes |
| 4 | No | 3 | 1 | 2 | Yes | 54 | L858R substitution | 1 | Erlotinib | 12.5 | 9 | No |
| 5 | No | 5 | 0 | 0 | Yes | 0 | L858R substitution | 2 | Afatinib | 12.5 | 16 | No |
| 6 | No | 1 | 4 | 3 | No | 0 | L858R substitution | 1 | Erlotinib | 10 | 7 | No |
| 7 | Yes | 2 | 3 | 1 | Yes | 38 | Rare mutation | 1 | Erlotinib | 7 | 5 | No |
| 8 | Yes | 3 | 4 | 0 | No | 26 | Exon 19 deletion | 1 | Erlotinib | 98 | 99 | No |
| 9 | Yes | 2 | 2 | 3 | No | 48 | L858R substitution | 2 | Afatinib | 22.5 | 30.5 | Yes |
| 10 | No | 1 | 4 | 0 | No | 32 | L858R substitution | 2 | Afatinib | 5 | 14 | Yes |
| 11 | No | 1 | 2 | 0 | No | 43 | Exon 19 deletion | 1 | Erlotinib | 53 | 60 | Yes |
| 12 | Yes | 2 | 1 | 1 | Yes | 20 | L858R substitution | 2 | Afatinib | 8,5 | 9.5 | No |
| 13 | No | 2 | 3 | 0 | Yes | 16 | Exon 19 deletion | 1 | Erlotinib | 10 | 9 | No |
| 14 | Yes | 2 | 4 | 1 | Yes | 31 | Exon 19 deletion | 1 | Erlotinib | 27 | 24 | No |
| 15 | No | 2 | 3 | 2 | Yes | 40 | Exon 19 deletion | 1 | Erlotinib | 15.5 | 16 | No |
| 16 | No | 2 | 2 | 1 | No | 40 | Exon 19 deletion | 2 | Afatinib | 19 | 15 | No |
| 17 | Yes | 1 | 4 | 2 | No | 5 | L858R substitution | 1 | Erlotinib | 16 | 16 | No |
| 18 | No | 2 | 0 | 3 | Yes | 0 | L858R substitution | 1 | Gefitinib | 4 | 9 | No |
| 19 | Yes | 3 | 2 | 2 | Yes | 45 | Exon 19 deletion | 1 | Erlotinib | 26 | 25 | No |
| 20 | Yes | 1 | 3 | 3 | Yes | 29 | Exon 19 deletion | 2 | Afatinib | 5.5 | 6 | No |
| 21 | No | 1 | 0 | 3 | Yes | 5 | L858R substitution | 1 | Erlotinib | 10 | 7 | No |
| 22 | Yes | 2 | 4 | 2 | Yes | 28 | L858R substitution | 1 | Gefitinib | 22 | 22 | No |
| 23 | No | 1 | 4 | 2 | Yes | 40 | Rare mutation | 2 | Afatinib | 9 | 6 | No |
| 24 | No | 1 | 4 | 3 | Yes | 43 | Rare mutation | 2 | Afatinib | 6 | 5.5 | No |
| 25 | No | 1 | 4 | 1 | Yes | 12 | L858R substitution | 2 | Afatinib | 19 | 19 | No |
| 26 | Yes | 3 | 3 | 0 | Yes | 15 | Rare mutation | 2 | Afatinib | 14.5 | 22 | No |
| 27 | No | 1 | 4 | 2 | Yes | 38 | L858R substitution | 1 | Erlotinib | 10 | 19 | No |
| 28 | Yes | 3 | 4 | 1 | Yes | 55 | L858R substitution | 1 | Gefitinib | 48 | 49 | No |
| 29 | No | 1 | 4 | 3 | Yes | 56 | L858R substitution | 1 | Gefitinib | 22 | 24 | No |
| 30 | No | 3 | 2 | 3 | Yes | 32 | L858R substitution | 2 | Afatinib | 5 | 26 | No |
| 31 | No | 1 | 0 | 0 | Yes | 0 | L858R substitution | 2 | Afatinib | 16.5 | 17 | No |
| 32 | No | 2 | 4 | 3 | Yes | 40 | L858R substitution | 1 | Gefitinib | 13.5 | 15 | No |
| 33 | No | 3 | 4 | 0 | Yes | 34 | Rare mutation | 2 | Afatinib | 17 | 25 | Yes |
| 34 | Yes | 1 | 4 | 0 | Yes | 0 | L858R substitution | 1 | Erlotinib | 5.5 | 10 | No |
| 35 | Yes | 1 | 4 | 3 | Yes | 38 | Exon 19 deletion | 1 | Gefitinib | 36 | 36 | No |
| 36 | Yes | 3 | 4 | 0 | Yes | 55 | L858R substitution | 2 | Afatinib | 12 | 21 | Yes |
| 37 | Yes | 1 | 4 | 3 | No | 45 | L858R substitution | 2 | Afatinib | 10 | 13.5 | No |
| 38 | No | 1 | 2 | 0 | No | 22 | L858R substitution | 1 | Erlotinib | 36 | 37 | Yes |
| 39 | No | 1 | 4 | 0 | No | 60 | L858R substitution | 1 | Erlotinib | 8 | 9 | Yes |
| 40 | No | 1 | 4 | 3 | No | 48 | Rare mutation | 1 | Erlotinib | 4 | 3.5 | No |
| 41 | No | 1 | 1 | 0 | Yes | 29 | L858R substitution | 2 | Afatinib | 15 | 16.5 | No |
